# Pre-clinical Positron Emission Tomography Reconstruction Algorithm Effect on Cu-64 ATSM Lesion Hypoxia

**DOI:** 10.4274/mirt.18189

**Published:** 2016-02-10

**Authors:** Bal Sanghera, Katie Wood, Luke I Sonoda, Andrew Gogbashian, Gerry Lowe, Andre Nunes, James Stirling, Chris Shepherd, Gwen Beynon, Wai Lup Wong

**Affiliations:** 1 Mount Vernon Hospital, Paul Strickland Scanner Centre, Northwood, UK; 2 Royal Surrey County Hospital, Clinic of Oncology, Guildford, UK

**Keywords:** Positron emission tomography scan, Cu-ATSM, hypoxia, animal, image reconstruction, image analysis

## Abstract

**Objective:**

Application of distinct positron emission tomography (PET) scan reconstruction algorithms can lead to statistically significant differences in measuring lesion functional properties. We looked at the influence of two-dimensional filtered back projection (2D FBP), two-dimensional ordered subset expectation maximization (2D OSEM), three-dimensional ordered subset expectation maximization (3D OSEM) without 3D maximum a posteriori and with (3D OSEM MAP) on lesion hypoxia tracer uptake using a pre-clinical PET scanner.

**Methods:**

Reconstructed images of a rodent tumor model bearing P22 carcinosarcoma injected with hypoxia tracer Copper-64-Diacetyl-bis (N4-methylthiosemicarbazone) (i.e. Cu-64 ATSM) were analyzed at 10 minute intervals till 60 minute post injection. Lesion maximum standardized uptake values (SUV_max_) and SUV_max_/background SUV_mean_ (T/B) were recorded and investigated after application of multiple algorithm and reconstruction parameters to assess their influence on Cu-64 ATSM measurements and associated trends over time.

**Results:**

SUV_max_SUV_max_ or T/B between 2D FBP, exhibited convergence for OSEM reconstructions while ANOVA results showed a significant difference in SUV_max_ or T/B between 2D FBP, 2D OSEM, 3D OSEM and 3D OSEM MAP reconstructions across all time frames. SUV_max_ and T/B were greatest in magnitude for 2D OSEM followed by 3D OSEM MAP, 3D OSEM and then 2D FBP at all time frames respectively. Similarly SUV_max_ and T/B standard deviations (SD) were lowest for 2D OSEM in comparison with other algorithms.

**Conclusion:**

Significantly higher magnitude lesion SUV_max_ and T/B combined with lower SD were observed using 2D OSEM reconstruction in comparison with 2D FBP, 3D OSEM and 3D OSEM MAP algorithms at all time frames. Results are SUV_max_ or T/B between 2D FBP, consistent with other published studies however more specimens are required for full validation.

## Abstract (tr)

**Amaç:**

Farklı pozitron emisyon tomografisi (PET) rekonstrüksiyon algoritmalarının uygulanması lezyonun fonksiyonel özelliklerinde istatistiksel olarak anlamlı farklılıklara neden olabilir. Bu çalışmada iki-boyutlu filtreli geri projeksiyon (2D FBP), iki-boyutlu düzenli subset expectation maksimizasyonu (2D OSEM), 3D maksimum a posteriori olmaksızın (3D OSEM) ve 3D maksimum a posteriori ile (3D OSEM MAP) üç-boyutlu düzenli subset expectation maksimizasyonu kullanımının pre-klinik bir PET tarayıcısı ile lezyon hipoksisini izleyen maddenin (tracer) tutulumu üzerine etkisi incelenmiştir.

**Yöntem:**

P22 karsinosarkomlu bir kemirgen tümör modelinde hipoksi izleyen maddenin Copper-64-Diacetyl-bis (N4-methylthiosemicarbazone) (Cu-64 ATSM) enjeksiyonu ile 60. dakikaya kadar her 10 dakikada bir elde edilen rekonstrükte görüntüler incelendi. Lezyonun maksimum standardize tutulum değeri (SUV_max_) ve SUV_max_/taban SUV_ortalama_ (T/B) değerleri kaydedildi, ve multipl algoritma ve rekonstrüksiyon parametresi uygulanmasından sonra bunların Cu-64 ATSM ölçümleri üzerindeki etkisi ve farklı zaman dilimlerindeki seyri araştırıldı.

**Bulgular:**

SUV_max_ OSEM rekonstrüksiyonları için konverjans gösterirken ANOVA sonuçları tüm zaman dilimlerinde 2D FBP, 2D OSEM, 3D OSEM ve 3D OSEM MAP rekonstrüksiyonları arasında SUV_max_ veya T/B de anlamlı farklılık tespit etti. En yüksek SUV_max_ ve T/B değerleri tüm zaman dilimlerinde 2D OSEM’de saptandı, bunu sırasıyla 3D OSEM MAP, 3D OSEM ve 2D FBP takip etti. Benzer şekilde SUV_max_ ve T/B standart deviasyonları (SD) diğer algoritmalara kıyasla 2D OSEM’de en düşüktü.

**Sonuç:**

2D OSEM rekonstrüksiyonu ile 2D FBP, 3D OSEM ve 3D OSEM MAP algoritmalarına kıyasla tüm zaman dilimlerinde anlamlı olarak daha yüksek lezyon SUV_max_ ve T/B ile birlikte daha düşük SD gözlemlendi. Sonuçlar daha önce yayınlanmış çalışmalarla uyumlu olmakla birlikte tam onaylama için daha fazla veri gerekmektedir.

## INTRODUCTION

Tumors can often grow rapidly outstripping the blood supply they depend upon leaving regions with low oxygenation. Known as hypoxia (1), this phenomenon can reduce the efficacy of treatment regimens e.g. chemotherapy and radiotherapy due to diminished blood supply typical of malignant lesions (2). Methods to map (3) hypoxic regions in lesions are encouraged as these areas can be offered modified treatment regimens to increase overall therapeutic efficacy (4).

Positron emission tomography (PET) is a quantitative imaging modality using spatial and temporal distributions of radiolabelled molecules (tracers) to identify functional tissue disease processes and to monitor these during treatment (5). A distinct advantage is its ability to detect small levels of tracer with high sensitivity without upsetting biological processes that occur naturally. PET depends upon radioactive labels attached to ligands or molecules of functional importance. Clinically the most common of these is F-18 attached to a glucose analogue F-18 fluorodeoxyglucose (FDG) to image glucose metabolism as many cancer cells exhibit enhanced glycolysis (6). In this context, small bore animal PET scanners offer advantages of accurate quantitative scanning in oncological (7,8) imaging applications leading to greater understanding of functional processes prior to potential translation to the clinic.

FDG PET has been applied to image hypoxia in cancer but other tracers are recommended (9) as glucose metabolism is considered a non-specific hypoxia marker. Copper-labeled ligands have shown promise in pre-clinical oncology imaging applications (10). Copper-64-Diacetyl-bis (N4-methylthiosemicarbazone) (Cu-64 ATSM) is considered as a more specific, hypoxia focused imaging agent with potential in radiotherapy treatment planning applications (11). Cu-64 ATSM yields relatively high tumor uptake, but in comparison with other hypoxia PET tracers, contradictory results and inconsistent correlation with immunohistochemistry hypoxia markers (12) are common. Besides variability driven by underlying biological processes, technological factors like image reconstruction algorithms also introduce uncertainty in SUV (13). As there is no optimal choice, algorithm selection can depend on requirements like quantitative accuracy, count-rate, maximising SUV or signal/noise (S/N) (14). It is recognised that changing software default parameters within a particular algorithm influences SUV and lesion detectability (15,16). Moreover, studies have been performed showing the effect of acquisition time on S/N using different reconstruction algorithms (17). The significance of accurate PET image reconstruction and semi-quantitative analysis in oncology should not be overlooked. For example, comparison of various algorithms resulted in clinically different dose distributions for proposed treatment regimes in one cancer study (18).

In our study, we present the effects of applying different image reconstruction algorithms to scans acquired from a rat tumor model at 10 min intervals upto an hour post injection of Cu-64 ATSM with a commercial pre-clinical PET scanner. Manufacturer supplied and widely used standard image reconstruction algorithms included 2D FBP, 2D OSEM, 3D OSEM and 3D OSEM MAP. In this setting, we compared the influence these image reconstruction algorithms make on SUV_max_ and T/B measurements that are commonly used to characterise lesions in hypoxia studies. Associated trends observed in these values at 10 min intervals are discussed with recommendations included over their combined impact on measuring hypoxia.

## MATERIALS AND METHODS

Cu-64 ATSM was produced in the Clinical PET Centre at St Thomas’s Hospital, London by a CTI RDS-112 cyclotron accelerating protons into a Ni-64 plated target, followed by subsequent separation and purification processes. This study was performed at a dedicated centre having considerable experience with P22 carcinosarcoma/BD9 rats whilst ensuring full regulatory compliance. Dynamic image data acquisition was initiated post injection of 35MBq Cu-64 ATSM using a MicroPET Focus 220 (Concorde microsystems incorporated). The scanner consisted of 48 detector rings with 504 LSO crystals per ring each crystal having dimensions 1.5 mm x1.5 mm x10 mm covering an axial field of view of approximately 7.7 cm. Scans were acquired on a single bed position over the lesion with a threshold window between 350 keV and 750 keV and 6ns timing window. Typical corrections were applied to validate efficacy of scans e.g. normalization, attenuation, arc, scatter etc. Transmission scans for attenuation correction over the area of interest were performed for 15 min using an integrated Co-57 source.

Hypoxia lesion characteristics were investigated on a single bed position following 10 min time frames acquired at 0-10 min, 10-20 min, 20-30 min, 30-40 min, 40-50 min and 50-60 min post injection of Cu-64 ATSM. Reconstructed scans consisted of (a) 2D FB, (b) 2D OSEM, (c) 3D OSEM and (d) 3D OSEM MAP algorithms using manufacturer supplied defaults. Reconstruction filters varied for analytical 2D FBP were Butterworth1 (b1), Butterworth2 (b2), Hamming, Hanning and with no filter for each time point investigated. All were performed at axial cutoff (Nyquist) 0.5 resulting in 30 distinct 2D FBP reconstructions.

Similarly, for iterative reconstruction 2D OSEM iterations (it) varied between 1, 2, 3, 4 and 5. For each iteration the following subsets (sub) were used 2, 4, 6, 8, 10, 16, 22 and 28 sub with Fourier rebinning employed resulting in 240 separate reconstructions. In the case of 3D OSEM 1, 2, 3, 4 and 5 it were employed with 9 sub for each time point investigated resulting in 30 measurements. In the case of 3D OSEM MAP 2 it and 9 sub was employed for the OSEM and 0, 2, 4, 6, 8, 10, 12, 14, 16 and 18 it for the MAP component; providing 60 reconstructions at different time points with a target FWHM of 1.5 mm.

**Figure 1:**
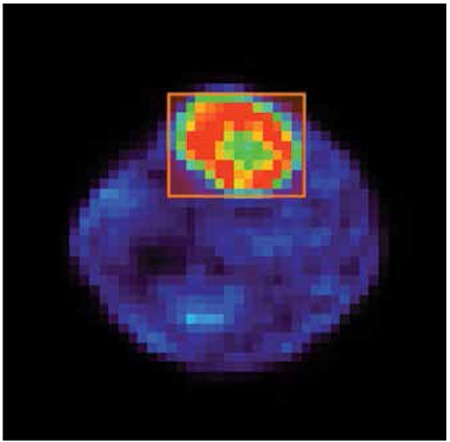
Image demonstrating rectangular volume of interest placement used to characterize the lesion imaged

These different parameters were used to study the effect of reconstruction algorithm settings on pre-clinical Cu-64 ATSM lesion hypoxia SUV_max_ and T/B for each 10 min time frame investigated. The same lesion or background defining volume of interest (VOI) was employed in the same location for all respective reconstructions to minimize variation arising from placement, Figure 1.

PSPP statistical software (19) was employed using one-way analysis of variance (ANOVA) with least significant difference (LSD) post hoc test to establish if differences between mean SUV_max_ acquired using different reconstructions methods for the same time frame were of statistical significance. This process was also applied to mean T/B again acquired using different reconstructions methods for the same respective time frame.

## RESULTS

### SUV_max_ Convergence

**Figure 2:**
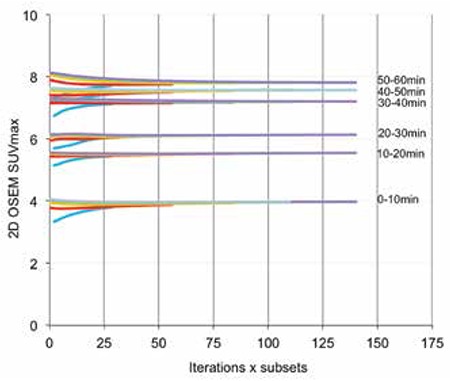
Two-dimensional ordered subset expectation maximization convergence for lesion SUVmax at 0-10 min, 10-20 min, 20-30 min, 30-40 min, 40-50 min and 50-60 min post injection

**Figure 3:**
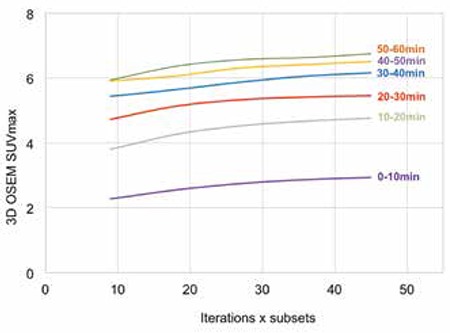
Three-dimensional ordered subset expectation maximization convergence with iteration for lesion SUVmax at 0-10 min, 10-20 min, 20-30 min, 30-40 min, 40-50 min and 50-60 min post injection

**Figure 4:**
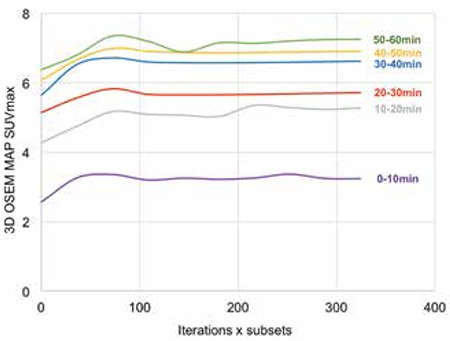
Three-dimensional ordered subset expectation maximization maximum a posteriori convergence with iteration for lesion SUVmax at 0-10 min, 10-20 min, 20-30 min, 30-40 min, 40-50 min and 50-60 min post injection

Figure 2 depicts lesion SUV_max_ measured using 2D OSEM reconstruction against the product of iterations and sub for different time frames. SUV_max_ were all found to approximate to a plateau at time points investigated endorsing convergence of 2D OSEM algorithm used. Figures 3 and 4 further support SUV_max_ convergence for 3D OSEM and 3D OSEM MAP reconstructions, respectively.

**Figure 5:**
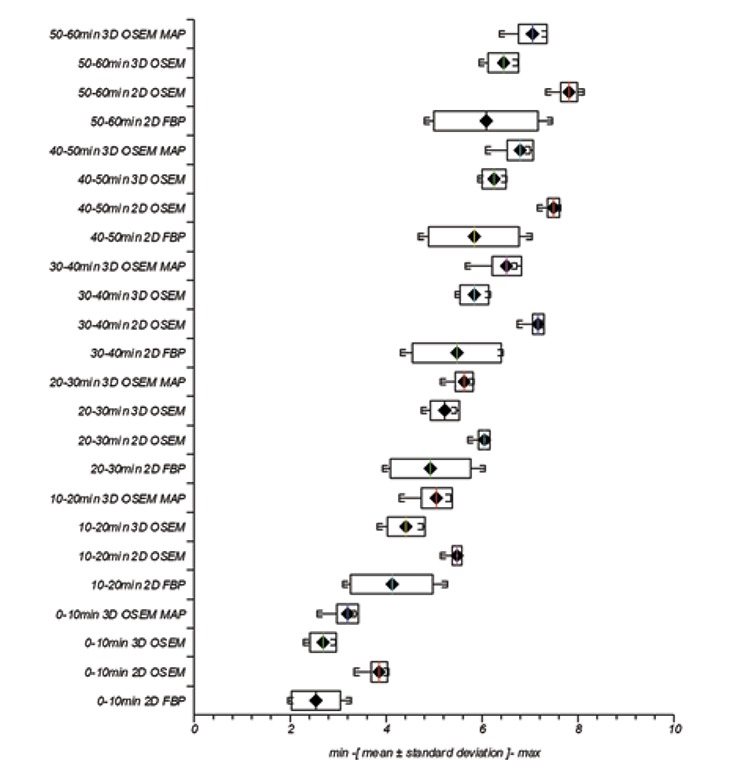
Variation in lesion SUVmax with two-dimensional filtered back projection, two-dimensional ordered subset expectation maximization, three-dimensional ordered subset expectation maximization and maximum a posteriori with different reconstruction parameters and at different time frames

Trends in variation of uptake measurements with reconstruction parameters are better characterized in box and whisker plots (depicting minimum, mean +/- standard deviation (SD) and maximum) for SUV_max_ Figure 5 respectively. The additional influence of scan timing on measured parameters at 0-10 min, 10-20 min, 20-30 min, 30-40 min, 40-50 min and 50-60 min is also seen.

### SUV_max_

For individual reconstruction algorithms, mean intra SUV_max_ was greatest in magnitude for 2D OSEM followed by 3D OSEM MAP, 3D OSEM and finally 2D FBP, Figure 5. This result was reflected at all acquisition times. Likewise for individual reconstruction algorithm intra SD of uptake measurements was least in magnitude for 2D OSEM followed by 3D OSEM MAP, 3D OSEM and finally 2D FBP. Again, this trend was reflected across all time frames.

### SUV_max_ One-Way ANOVA

Statistical analysis revealed that mean SUV_max_ acquired at 0-10 min time frame exhibited a significant difference (F(3.60)=4.7, p=0.0) between reconstruction groups 2D FBP, 2D OSEM, 3D OSEM and 3D OSEM MAP. This result was repeated at 10-20 min (F(3.60)=4.12, p=0.0), 20-30 min (F(3.60)=2.81, p=0.0), 30-40 min (F(3.,60)=6.67, p=0.0), 40-50 min (F(3.60)=6.32, p=0.0) and 50-60 min (F(3.60)=7.2, p=0.0). It can be seen in Table 1 that reconstruction groups display significant differences in mean SUV_max_ within each respective time frame besides 2D FBP with 3D OSEM. It is observed the magnitude of mean SUV_max_ for 3D OSEM >2D FBP at 0-10 min, 10-20 min, 20-30 min, 30-40 min and 50-60 min respectively. Non-statistically significant results are denoted with*.

### T/B

**Figure 6:**
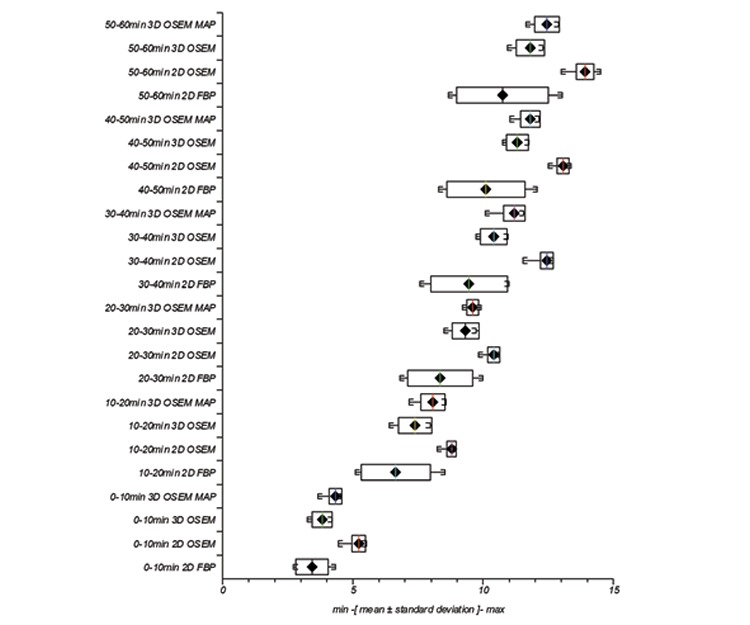
Variation in lesion T/B with two-dimensional filtered back projection, two-dimensional ordered subset expectation maximization, three-dimensional ordered subset expectation maximization and maximum a posteriori with different reconstruction parameters and at different time frames

At each acquisition time, individual reconstruction algorithm’s mean T/B was greatest in magnitude for 2D OSEM followed by 3D OSEM MAP, 3D OSEM and finally 2D FBP, Figure 6. Equally, individual reconstruction algorithm’s intra SD for uptake measurements was least in magnitude for 2D OSEM followed by 3D OSEM MAP, 3D OSEM and finally 2D FBP across all time points.

### T/B One-Way ANOVA

Statistical analysis revealed that mean T/B derived at 0-10 min time frame exhibited a significant difference (F(3.60)=8.03, p=0.0) between reconstruction groups 2D FBP, 2D OSEM, 3D OSEM and 3D OSEM MAP. This result was repeated at 10-20 min (F(3.60)=9.36, p=0.0), 20-30 min (F(3.60)=8.19, p=0.0), 30-40 min (F(3.60)=19.85, p=0.0), 40-50 min (F(3.60)=18.16, p=0.0) and 50-60 min (F(3.60)=22.33, p=0.0).

It can be seen in Table 2 that reconstruction groups display significant differences in mean T/B within each respective time frame besides 3D OSEM with 3D OSEM MAP at 20-30 min and 40-50 min. It is observed the magnitude of mean T/B for 3D OSEM MAP >3D OSEM at 0-10 min, 10-20 min, 20-30 min, 30-40 min and 50-60 min respectively. Non-statistically significant results are denoted with*.

## DISCUSSION

Pre-clinical PET scanner studies provide an opportunity to investigate accurate, quantitative, functional properties of lesions in preparation of potential clinical trials. In this study we investigated the effect of available and widely used reconstruction algorithms on Cu-64 ATSM hypoxia characteristics of a rodent tumor rather than investigating general biochemical uptake mechanisms or phantom scan data. Furthermore, in relation to other animal tumors, this P22 model was more oxic, solid and relatively large, thereby more relevant to a human tumor model.

Additional reconstructions had to be performed retrospectively following relocation of the scanner, with associated dedicated software and hardware to another imaging centre. Siemens Imaging Oxford kindly assisted with supply of reconstruction software and default supplementary files necessary to perform these locally at our site. A networked Intel Pentium 4 CPU, 3.4 GHz, 1 GB RAM 80 GB hard drive PC running Windows XP 64 bit operating system was identified as a compatible machine and painstakingly configured to perform reconstructions. This platform enabled complex reconstruction e.g. 2D FBP, 2D OSEM 5 it 28 sub, 3D OSEM 5 it and 3D OSEM MAP 2 it 18 sub algorithms to be completed in approximately 10 min, 1 hr, 44.5 hr and 90 hr respectively.

All reconstruction algorithms showed increased magnitude of mean SUV_max_ and mean T/B with time frame measured, reflecting known uptake characteristics of Cu-64 ATSM for hypoxia measurements (20), thus offering some level of assurance that reconstruction algorithms were functioning appropriately. In order to assist characterizing reconstruction effects using different algorithms, we identified general trends from SUV_max_ and T/B results acquired at time frames specified. Figures 5 and 6 show a trend in mean value magnitudes of SUV_max_ and T/B where 2D OSEM >3D OSEM MAP >3D OSEM >2D FBP at different time frames. Similarly with measurements of SD for mean SUV_max_ and mean T/B, Figures 5 and 6, we see a trend where 2D OSEM <3D OSEM or 3D OSEM MAP <2D FBP at different time frames.

Larger SD across reconstructions observed with FBP algorithm for mean SUV_max_ and mean T/B may reflect limitations of this algorithm (13), arising from applying available defaults and possible low count-rate statistics. As expected for each respective time frame and within each respective reconstruction algorithm, mean T/B >corresponding mean SUV_max_ and the difference was statistically significant (p<0.0001 in all cases) (21).

It may be argued amongst the reconstruction parameters available and consequently used with our imaging system that 2D OSEM potentially offers a good compromise for improved imaging. For all time frames investigated, 2D OSEM generated SUV_max_ and T/B consistently demonstrated relatively large magnitude mean values and exhibited less SD in comparison with other reconstruction algorithms for parameters used. Our endorsement of 2D OSEM image reconstruction agrees with another similar study using a newer generation scanner (13).

Our aim was to look at differences introduced by multiple algorithms with various available default parameters and observe trends in SUV_max_ and T/B measurements often used to characterize lesions. Limitations to the study arose from lack of objective gold standard with which to compare results using different standard image reconstruction algorithms in the genuine rat tumor investigated. Furthermore, scans were reconstructed at specific time periods with data available from one sacrificed animal only. Hence, results presented should not be extrapolated beyond this remit without further studies. However, there is no reason to doubt that the outcome of this study is not representative of a rat P22 tumor model and results presented are supported by others (13).

## CONCLUSION

Typical image reconstruction algorithms and parameters were compared for lesion SUV_max_ or T/B in 10 min time frames 0-10 min, 10-20 min, 20-30 min, 30-40 min and 50-60 min time points post injection following dynamic hypoxia scanning of a rat tumor with Cu-64 ATSM. It was observed that 2D OSEM in comparison with 2D FBP, 3D OSEM and 3D OSEM MAP reconstructions represented the highest magnitude SUV_max_ and T/B, combined with the lowest SD respectively for the hypoxic lesion studied. Differences between reconstruction algorithms in the vast majority of cases were statistically significant at time points measured. More specimens are required for full validation though results are consistent with other published studies.

## References

[1] Leach RM, Treacher DF (1998). Oxygen transport-2. Tissue hypoxia. BMJ.

[2] Vaupel P, Mayer A (2007). Hypoxia in cancer: significance and impact on clinical outcome. Cancer Metastasis Rev.

[3] Krohn KA, Link JM, Mason RP (2008). Molecular imaging of hypoxia. J Nucl Med.

[4] Wilson WR, Hay MP (2011). Targeting hypoxia in cancer therapy. Nat Rev Cancer.

[5] Carlier T, Bailly C (2015). State-of-the-art and recent advances in quantification for therapeutic follow-up in oncology using PET. Front Med (Lausanne).

[6] Warburg O (1956). On the origin of cancer cells. Science.

[7] Yao R, Lecomte R, Crawford ES (2012). Small-animal PET: what is it, and why do we need it?. J Nucl Med Technol.

[8] Kuntner C, Stout D. Quantitative preclinical PET imaging: opportunities and challenges.

[9] Dierckx RA (2008). FDG uptake, a surrogate of tumour hypoxia?. Eur J Nucl Med Mol Imaging.

[10] Niccoli Asabella A, Cascini GL, Altini C, Paparella D, Notaristefano A, Rubini G (2014). The copper radioisotopes: a systematic review with special interest to 64Cu. Biomed Res Int.

[11] Clausen MM, Hansen AE, Lundemann M, Hollensen C, Pommer T, Kristensen AT, Kjær A, McEvoy FJ, Engelholm SA (2014). Dose painting based on tumor uptake of Cu-ATSM and FDG: a comparative study. Radiat Oncol.

[12] Carlin S, Zhang H, Reese M, Ramos NN, Chen Q, Ricketts SA (2014). A comparison of the imaging characteristics and microregional distribution of 4 hypoxia PET tracers. J Nucl Med.

[13] Yu AR, Kim JS, Kang H, Lim SM (2015). Comparison of reconstruction methods and quantitative accuracy in Siemens Inveon PET scanner. J Inst.

[14] Thorwarth D, Mönnich D, Zips D (2013). Methodological aspects on hypoxia PET acquisition and image processing. Q J Nucl Med Mol Imaging.

[15] Defrise M, Kinahan P E, Michel CJ, Bailey DL, Townsend DW, Valk PE, Maisey MN (2005). Image reconstruction algorithms PET. Positron emission tomography.

[16] Schaefferkoetter J, Casey M, Townsend D, El Fakhri GE (2013). Clinical impact of time-of-flight and point response modeling in PET reconstructions: a lesion detection study. Phys Med Biol.

[17] Morey AM, Kadrmas DJ (2013). Effect of varying number of OSEM subsets on PET lesion detectability. J Nucl Med Technol.

[18] Knudtsen IS, Elmpt W, Ollers M, Malinen E (2014). Impact of PET reconstruction algorithm and threshold on dose painting of non-small cell lung cancer. Radiother Oncol.

[19] Plaff B, Darrington J, GNU PSPP. Version 0.8.4.

[20] Bayly SR, King RC, Honess DJ, Barnard PJ, Betts HM, Holland JP, Hueting R, Bonnitcha PD, Dilworth JR, Aigbirhio FI, Christlieb M (2008). In vitro and in vivo evaluations of a hydrophilic 64Cu bis(thiosemicarbazonato)-glucose conjugate for hypoxia imaging. J Nucl Med.

[21] Knight JC, Wuest M, Saad FA, Wang M, Chapman DW, Jans HS, Lapi SE, Kariuki BM, Amoroso AJ, Wuest F (2013). Synthesis, characterisation and evaluation of a novel copper-64 complex with selective uptake in EMT-6 cells under hypoxic conditions. Dalton Trans.

